# Risk factors for postoperative delirium on oxygen delivery-guided perfusion

**DOI:** 10.1186/s13019-022-01938-z

**Published:** 2022-08-20

**Authors:** Hiroshi Mukaida, Satoshi Matsushita, Yuki Minami, Go Sato, Masato Usuba, Rinako Kondo, Tohru Asai, Atsushi Amano

**Affiliations:** 1 Department of Clinical Engineering, Faculty of Medical Science, Juntendo University, Chiba, Japan; 2 Department of Clinical Engineering, Juntendo University Hospital, Tokyo, Japan; 3 Department of Cardiovascular Surgery, Faculty of Medicine, Juntendo University, Hongo 2-1-1, Bunkyo-ku, Tokyo, 113-8421 Japan

**Keywords:** Cardiopulmonary bypass, Optimal perfusion, Goal directed perfusion, Oxygen delivery, Hemodilution

## Abstract

**Background:**

Studies have demonstrated the efficacy of oxygen delivery-guided perfusion (ODGP) in preventing postoperative acute kidney injury, but the benefit of ODGP for delirium has not been confirmed. We retrospectively investigated the risk factors for postoperative delirium in patients who underwent ODGP (with oxygen delivery index [DO_2_i] > 300 mL/min/m^2^).

**Methods:**

Consecutive patients who underwent on-pump cardiovascular surgery with ODGP from January 2018 to December 2020 were retrospectively analyzed. In addition to examining patients’ DO_2_i during cardiopulmonary bypass (CPB), we quantified the two primary DO_2_ components-hematocrit (Hct) and pump flow. Delirium was defined based on the Intensive Care Delirium Screening Checklist (ICDSC). Patients were divided into three groups: no delirium (ICDSC score = 0), subsyndromal delirium (ICDSC score = 1–3), and clinical delirium (ICDSC score ≥ 4).

**Results:**

Multivariate analysis identified only the number of red blood cell (RBC) units transfused, intubation time, and the cumulative time below the Hct threshold of 25% as predictive factors of postoperative delirium. Although patients with higher ICDSC scores had greater hemodilution during CPB, ODGP resulted in a higher pump flow, and DO_2_i was maintained above 300 mL/min/m^2^, with no significant difference between the three groups.

**Conclusions:**

A low Hct level during CPB with ODGP, the number of RBC units transfused, and intubation time were associated with postoperative delirium. Further investigations are needed to determine the ability of ODGP to prevent low Hct during CPB.

## Background

Postoperative delirium is a common complication after cardiac surgery, with a reported frequency of 3% to 31% [1]. Postoperative delirium is associated with short- and long-term adverse outcomes, including longer length of hospital stay, greater prevalence of falls after surgery, and postoperative stroke and death [2, 3]. Moreover, strategies to prevent these adverse outcomes of postoperative delirium are lacking [4]. Although postoperative delirium has been attributed to age, diabetes, pre-existing cerebrovascular disease, peripheral vascular disease, blood transfusion, renal insufficiency, and postoperative ventilation time [1, 3], many of these risk factors are unmodifiable.

Recently, many studies have directly examined the relationship between oxygen delivery (DO_2_) during cardiopulmonary bypass (CPB) and postoperative acute kidney injury (AKI) [5–11]. Moreover, the relationship between low DO_2_ during CPB and postoperative delirium has also been reported [12], and CPB management using DO_2_ as an index of perfusion flow has received increased attention as a new concept regarding optimal perfusion flow. In a previous study, we reported that oxygen delivery-guided perfusion (ODGP), preventing a DO_2_ index (DO_2_i) of < 300 mL/min/m^2^ during CPB, reduced the incidence of postoperative AKI.^13^ Since then, we have performed ODGP (maintaining DO_2_i > 300 mL/min/m^2^ through pump flow adjustments during CPB) to prevent postoperative AKI. Studies have demonstrated the efficacy of ODGP in preventing postoperative AKI [10, 13], but the benefits of ODGP for delirium have not been confirmed. We aimed to retrospectively investigate the risk factors for postoperative delirium in patients who underwent ODGP (with DO_2_i > 300 mL/min/m^2^).

## Methods

### Patient population

This retrospective study included 216 adult patients who underwent on-pump cardiovascular surgery with ODGP at Juntendo University Hospital from January 2018 to December 2020. The exclusion criteria were emergency surgery, requirement for circulation arrest, presence of severe chronic kidney disease (estimated glomerular filtration rate [eGFR] < 30 mL/min/1.73 m^2^) preoperatively, discontinuation of ODGP because of poor venous drainage, and incomplete preoperative or postoperative data. The study was approved by the institutional review board of Juntendo University Hospital (17–184).

### Anesthesia and cardiopulmonary bypass procedure

Anesthesia was induced with midazolam, fentanyl, and rocuronium, and maintained with sevoflurane and remifentanil before CPB, and with propofol and remifentanil during and after CPB. After the surgery, remifentanil was discontinued, and fentanyl was administered. Propofol was continued until extubation in the intensive care unit (ICU).

CPB was performed under normothermic conditions (≥ 35 °C). We maintained a DO_2_i value of > 300 mL/min/m^2^ through pump flow adjustments during CPB. The minimum pump flow was set as 2.6 L/min/m^2^. Red blood cell (RBC) transfusion during CPB was considered when hemoglobin (Hb) values were < 7 g/dL. Roller pumps (LivaNova, Munich, Germany) were used for CPB. Mean arterial pressure (MAP) levels of 60 mmHg were maintained with intermittent intravenous phenylephrine administration. The CDI® Blood Parameter Monitoring System 500 (Terumo, Tokyo, Japan) was recalibrated every 20 min using ABL800 FLEX blood gas analyzer (Radiometer Medical ApS, Denmark). Oxygen-related measurements, including hematocrit (Hct) and DO_2_i values, were performed using the LivaNova CONNECT data management system (LivaNova), which recorded data every 20 s.

### Data collection and definitions

The collected demographic data included age, sex, body habitus, comorbidities, previous transient ischemic attack/cerebral vascular accident (TIA/CVA), left ventricular ejection fraction, eGFR, preoperative Hct, preoperative albumin, and Euro SCORE II.

Intraoperative variables included the type of surgery, pump time, nadir DO_2_i, median DO_2_i, area under the curve below the 300 mL/min/m^2^ DO_2_i threshold (AUC < DO_2_i^300^), cumulative time below the 300 mL/min/m^2^ DO_2_i threshold (time < DO_2_i^300^), median perfusion index (PI), nadir Hct, AUC below the 60 mmHg MAP threshold (AUC < MAP^60^), cumulative time below the 60 mmHg MAP threshold (time < MAP^60^), number of RBC units transfused, nadir cerebral oxygen saturation (ScO_2_) during CPB, AUC below the 80% of baseline ScO_2_ (AUC < ScO_2_^80%bas^), and cumulative time below the 80% of baseline ScO_2_ (time < ScO_2_^80%bas^). These nadir points were defined as the lowest points during the measurement. Postoperative variables included intubation time, systolic blood pressure, blood glucose, sodium, and chloride. Systolic blood pressure, blood glucose, sodium, and chloride data were collected the day after surgery. DO_2_i was calculated according to the equation: DO_2_i (mL/min/m^2^) = pump flow (L/min) × [Hct/2.94 (g/dL) × 1.36 × SaO_2_ (%) + PaO_2_ (mmHg) × 0.003] × 10 / BSA (m^2^), where SaO_2_ is oxygen saturation in the arterial blood, PaO_2_ is partial pressure of oxygen in the arterial blood, and BSA is body surface area.

In addition to examining the patients’ DO_2_ during CPB, we quantified the two primary DO_2_ components – Hct and pump flow. To quantify hemodilution during CPB, the AUC below the 21%, 23%, and 25% Hct thresholds (AUC < Hct^21^, AUC < Hct^23^, and AUC < Hct^25^) and the cumulative time below the 21%, 23%, and 25% Hct thresholds (time < Hct^21^, time < Hct ^23^, and time < Hct^25^) were calculated. Similarly, to quantify pump flow during CPB, the AUC below the 2.6, 2.7, and 2.8 L/min/m^2^ PI thresholds (AUC < PI^2.6^, AUC < PI^2.7^, and AUC < PI^2.8^) and the cumulative time below the 2.6, 2.7, and 2.8 L/min/m^2^ PI thresholds (time < PI^2.6^, time < PI^2.7^, and time < PI^2.8^) were calculated.

### Definition of delirium

Delirium was defined based on the Intensive Care Delirium Screening Checklist (ICDSC) [14]. ICDSC is an eight-item checklist that produces a score between 0 and 8. The ICDSC scores were calculated once every 8 h by nurses. The score during ICU stay within 48 h postoperatively was categorized into three groups. No delirium was defined as the non-manifestation of any of the eight items (ICDSC score 0). Subsyndromal delirium was defined as the manifestation of some of the eight items (ICDSC score 1–3) without reaching the diagnostic criteria for clinical delirium. Clinical delirium was defined as the manifestation of at least four of the eight items (ICDSC score ≥ 4). Patients who did not permit evaluation with the ICDSC throughout their ICU stay (postoperative 48 h) were excluded.

### Statistical analysis

Continuous and categorical variables are expressed as mean ± standard deviation (SD) or median (interquartile range) and numbers (%), respectively. Continuous variables were compared between the three groups using a one-way analysis of variance (ANOVA) followed by Tukey’s honestly significant difference test if the one-way ANOVA was significant. For continuous variables with skewed distribution, the nonparametric Kruskal–Wallis test was used, followed by the Steel–Dwass test in the case of significant findings. Categorical variables were analyzed using the Pearson chi-squared test. These tests were used to comprehensively evaluate the associations between perioperative variables and ICDSC scores; thus, candidate predictors of delirium were selected. Subsequently, a multivariate ordered logistic regression analysis was performed to identify significant predictors of delirium. Moreover, a multivariate ordered logistic regression model was built based on previously reported independent risk factors (age, diabetes, previous TIA/CVA, eGFR, preoperative albumin, number of RBC units transfused, intubation time, systolic blood pressure, blood glucose, and sodium) for delirium [1, 15–17]. Statistical significance was set at *p* < 0.05. The Holm method was used to adjust the p-values for multiple comparisons of categorical variables. To make the presentation simpler, we compared the p-value adjusted with 0.05 to determine whether a particular test result was statistically significant after adjustment. All analyses were performed using JMP14 software (SAS Institute. Cary, NC, USA).

## Results

### Patient characteristics

The characteristics of the 225 patients are shown in Table 1. Those in the study population who were diagnosed with no delirium, subsyndromal delirium, and clinical delirium during the postoperative 48 h were 53 (23.6%), 163 (72.4%), and 9 (4.0%), respectively. There were significant differences in BSA, diabetes, preoperative Hct, and Euro SCORE II values. Patients who developed clinical delirium were more likely to have smaller BSA, diabetes, lower preoperative Hct, and higher Euro SCORE II.

**Table 1:** Demographic characteristics of patients in the groups with no delirium, subsyndromal delirium, and clinical delirium

Variable	No delirium n = 53 (23.6%)	Subsyndromal delirium n = 163 (72.4%)	Clinical delirium n = 9 (4.0%)	*P* value			
				Overall	ND vs. SD	ND vs. CD	SD vs. CD
Age (years)	64.7 ± 14.2	67.5 ± 12.1	71.9 ± 8.7	0.193			
Male	35 (66.0%)	97 (59.5%)	4 (44.4%)	0.424			
BSA (m^2^)	1.68 ± 0.19	1.62 ± 0.19	1.51 ± 0.14	0.014	0.06	0.033	0.246
BMI (kg/m^2^)	23.6 ± 3.6	22.7 ± 3.2	21.8 ± 2.3	0.163			
Hypertension	26 (49.1%)	79 (48.5%)	6 (66.7%)	0.568			
Diabetes	8 (15.1%)	19 (11.7%)	4 (44.4%)	0.020	0.511	0.078	0.015
Peripheral vascular disease	0	1 (0.6%)	0	0.826			
Previous TIA/CVA	11 (20.8%)	30 (18.4%)	4 (44.4%)	0.162			
LVEF (%)	62.9 ± 10.7	62.5 ± 11.0	62.7 ± 12.8	0.976			
eGFR (mL/min/1.73m^2^)	71.0 ± 19.7	69.8 ± 19.7	56.9 ± 20.4	0.138			
Preoperative Hct (%)	38.9 ± 4.3	38.4 ± 4.8	34.0 ± 4.4	0.016	0.793	0.012	0.018
Preoperative albumin (g/dL)	4.0 ± 0.5	4.0 ± 0.3	3.9 ± 0.6	0.816			
Euro SCORE II	1.50 (0.85–3.06)	1.95 (1.01–3.40)	6.68 (2.33–11.50)	0.005	0.471	0.006	0.01

Continuous and categorical data are expressed as means ± standard deviations or medians (interquartile ranges), and numbers (%), respectively

*BMI* body mass index, *BSA* body surface area, *CD* clinical delirium, *CVA* cerebral vascular accident, *eGFR* estimated glomerular filtration rate, *Euro SCORE* European System for Cardiac Operative Risk Evaluation, *Hct* hematocrit, *LVEF* left ventricular ejection fraction, *ND* no delirium, *SD* subsyndromal delirium, *TIA* transient ischemic attack

### Perioperative data

Table 2 shows the perioperative data in the three groups. Although no significant differences were found in nadir DO_2_i, median DO_2_i, AUC < DO_2_i^300^, and time < DO_2_i^300^, median PI, nadir Hct, number of RBC units transfused were significantly different between the three groups. Patients with higher ICDSC scores tended to have a higher median PI, lower nadir Hct, a greater number of RBC units transfused, higher blood glucose, and higher sodium.

**Table 2:** Perioperative data in the groups with no delirium, subsyndromal delirium, and clinical delirium

Variable	No delirium (n = 53)	Subsyndromal delirium (n = 163)	Clinical delirium (n = 9)	*P* value			
				Overall	ND vs. SD	ND vs. CD	SD vs. CD
Surgical procedure				0.986			
Valve	41 (77.4%)	127 (77.9%)	8 (88.9%)				
Valve + TA replacement	6 (1.3%)	20 (12.3%)	1 (11.1%)				
TA replacement	1 (1.9%)	3 (1.8%)	0				
Adult congenital	1 (1.9%)	5 (3.1%)	0				
Cardiac tumor	4 (7.6%)	8 (4.9%)	0				
Pump time (min)	127 (105–167)	128 (103–177)	173 (135–213)	0.110			
Nadir DO_2_i (mL/min/m^2^)	292.2 ± 28.4	286.1 ± 28.5	278.3 ± 18.6	0.251			
Median DO_2_i (mL/min/m^2^)	341.8 ± 20.9	339.9 ± 24.4	349.1 ± 25.9	0.488			
AUC < DO_2_i^300^	42 (0–159)	68 (0–516)	265 (6–658)	0.246			
Time < DO_2_i^300^ (min)	2.3 (0–6.8)	2.3 (0–11.7)	8 (0.5–10.7)	0.519			
Median PI (L/min/m^2^)	2.72 (2.63–2.80)	2.78 (2.68–2.85)	2.86 (2.69–2.92)	0.012	0.025	0.118	0.415
Nadir Hct (%)	23 (21–24)	22 (21–23)	21 (20.5–22)	0.048	0.096	0.161	0.481
AUC < MAP^60^	571 (285–795)	620 (348–1151)	368 (235–729)	0.065			
Time < MAP^60^ (min)	28.9 ± 31.5	31.7 ± 25.6	24.4 ± 19.7	0.635			
Units of RBC transfused (u)	2.1 ± 4.1	2.4 ± 3.4	6.6 ± 3.7	0.003	0.872	0.003	0.003
Nadir ScO_2_ (%)	51 (43–58)	50 (42–56)	47.5 (41.5–55)	0.519			
AUC < ScO_2_^80%bas^	0 (0–4.5)	0 (0–28.6)	2.3 (0.0–114.9)	0.247			
Time < ScO_2_^80%bas^ (min)	0 (0–2.4)	0 (0–15.2)	1.6 (0.1–40.8)	0.094			
Intubation time (h)	6.7 (5.0–9.4)	6.2 (4.2–9.4)	15.2 (4.8–57.3)	0.123			
Systolic blood pressure (mmHg)	118.7 ± 18.9	115.1 ± 21.1	123.9 ± 26.2	0.604			
Blood glucose (mg/dL)	128 (110–148)	135 (118–152)	144 (131–187)	0.039	0.465	0.285	0.417
Sodium (mEq/L)	141.9 ± 3.4	142.7 ± 0.3	144.8 ± 2.7	0.026	0.283	0.045	0.162
Chloride (mEq/L)	107.0 ± 4.3	107.3 ± 4.2	109.2 ± 4.3	0.321			

Continuous and categorical data are expressed as means ± standard deviations or medians (interquartile ranges), and numbers (%), respectively. Nadir points were defined as the lowest points during measurement. The type of thoracic aortic replacement was ascending aortic replacement (excluded circulatory arrest under hypothermia). Systolic blood pressure, blood glucose, sodium, and chloride data were collected the day after surgery. AUC < MAP^60^, area under the curve below the 60 mmHg mean arterial pressure thresholds; AUC < DO_2_i^300^, area under the curve below the 300 mL/min/m^2^ oxygen delivery thresholds; AUC < ScO_2_^80%bas^, area under the curve below the 80% of baseline cerebral oxygen saturation

Time < MAP^60^, cumulative time below the 60 mmHg mean arterial pressure thresholds; Time < DO_2_i^300^, cumulative time below the 300 mL/min/m^2^ oxygen delivery thresholds; Time < ScO_2_^80%bas^, cumulative time below the 80% of baseline cerebral oxygen saturation

*CD* clinical delirium, DO_2_i oxygen delivery index, *Hct* hematocrit, *ND* no delirium, *PI* perfusion index, *RBC* red blood cell, ScO_2_ cerebral oxygen saturation, *SD* subsyndromal delirium, *TA* thoracic aortic

The relationships between the two primary DO_2_ components during CPB and delirium are presented in Table 3. Time < Hct^23^, AUC < Hct^25^, and time < Hct^25^ were significantly different between the three groups. Patients with higher ICDSC scores were more likely to have greater hemodilution during CPB. However, there was no significant difference in pump flow between the three groups, but there was a trend toward higher pump flow in those with higher ICDSC scores.

**Table 3:** The two primary DO_2_ components during CPB and delirium

Variable	No delirium (n = 53)	Subsyndromal delirium (n = 163)	Clinical delirium (n = 9)	*P* value			
				Overall	ND vs. SD	ND vs. CD	SD vs. CD
AUC < Hct^21^	0 (0–0)	0 (0–0)	0 (0–6.5)	0.268			
Time < Hct^21^ (min)	0 (0–0)	0 (0–0)	0 (0–2.2)	0.308			
AUC < Hct^23^	0 (0–33.5)	6 (0–60)	35 (10.5–122)	0.055			
Time < Hct^23^ (min)	0 (0–8.2)	2 (0–16)	9.3 (2.7–20.9)	0.049	0.096	0.117	0.540
AUC < Hct^25^	60 (17.5–221.5)	154 (22–421)	205 (94.5–562.5)	0.048	0.084	0.153	0.587
Time < Hct^25^ (min)	18.3 (5.5–43.9)	31.7 (7–75.3)	58 (23.3–125.7)	0.021	0.081	0.055	0.271
AUC < PI^2.6^	0.79 (0.26–3.88)	0.44 (0.04–2.50)	0.12 (0.02–2.90)	0.223			
Time < PI^2.6^ (min)	3.3 (1.7–26.8)	3 (0.7–11.0)	5 (0.9–10.2)	0.344			
AUC < PI^2.7^	8.6 (2.3–22.4)	3.8 (1.3–14.3)	4.0 (2.0–20.4)	0.097			
Time < PI^2.7^ (min)	41 (14.2–99)	20.3 (7.3–66)	15.3 (8.8–80.9)	0.086			
AUC < PI^2.8^	30.6 (13.3–61)	16.1 (6.5–42.1)	10.8 (5.7–48.2)	0.081			
Time < PI^2.8^ (min)	91 (42.3–132.5)	62.7 (21.7–116)	44.7 (14.7–134)	0.065			

AUC < Hct^21^, area under the curve below the 21% hematocrit thresholds; AUC < Hct^23^, area under the curve below the 23% hematocrit thresholds; AUC < Hct^25^, area under the curve below the 25% hematocrit thresholds; AUC < PI^2.6^, area under the curve below the 2.6 perfusion index thresholds; AUC < PI^2.7^, area under the curve below the 2.7 perfusion index thresholds; AUC < PI^2.8^, area under the curve below the 2.8 perfusion index thresholds; CD, clinical delirium; CPB, cardiopulmonary bypass; DO_2_, oxygen delivery; Hct, hematocrit; ND, no delirium; SD, subsyndromal delirium; Time < Hct^21^, cumulative time below the 21% hematocrit thresholds; Time < Hct^23^, cumulative time below the 23% hematocrit thresholds; Time < Hct^25^, cumulative time below the 25% hematocrit thresholds; Time < PI^2.6^, cumulative time below the 2.7 perfusion index thresholds; Time < PI^2.8^, cumulative time below the 2.8 perfusion index thresholds; Time < PI^2.8^, cumulative time below the 2.8 perfusion index thresholds

### Perioperative variables predicting delirium

Twelve perioperative variables (BSA, diabetes, preoperative Hct, Euro SCORE II, median PI, nadir Hct, units of RBC transfused, blood glucose, sodium, time < Hct^23^, AUC < Hct^25^, and time < Hct^25^) were significantly associated with delirium (*p* < 0.05 each). Variables related to hemodilution, including nadir Hct, time < Hct^23^, AUC < Hct^25^, and time < Hct^25^, were correlated with each other; thus, time < Hct^25^ was selected as a candidate predictor of delirium because time < Hct^25^ was more significantly associated with delirium compared to nadir Hct, time < Hct^23^, and AUC < Hct^25^. Multivariate ordered logistic regression analysis for the ICDSC score was performed by entering nine variables, including time < Hct^25^, into the model. This analysis revealed that time < Hct^25^ remained a significant independent predictor of the ICDSC score (*p* = 0.019) (Table 4, model 1). In model 2, only the number of RBC units transfused, intubation time, and time < Hct^25^ were independently associated with the ICDSC score (*p* = 0.018, *p* = 0.031, and *p* = 0.015, respectively) (Table 4, model 2).

**Table 4:** Multivariate ordered logistic regression analysis for ICDSC score

Variable	Coefficient	Standard error	Chi-Square	*P* value
*Model 1*				
BSA (m^2^)	0.84	1.27	0.44	0.508
Diabetes	− 0.52	0.33	2.47	0.116
Preoperative Hct (%)	− 0.05	0.05	1.07	0.301
Euro SCORE II	− 0.11	0.07	2.91	0.088
Median PI (L/min/m^2^)	− 2.09	1.42	2.16	0.141
Number of RBC units transfused (u)	− 0.12	0.08	2.05	0.153
Blood glucose (mg/dL)	− 0.00	0.01	0.04	0.526
Sodium (mEq/L)	− 0.01	0.06	0.01	0.907
Time < Hct^25^ (min)	− 0.01	0.00	5.51	0.019
Overall model fit		AIC = 217.7		0.002
*Model 2*				
Age (years)	− 0.01	0.02	0.36	0.547
Diabetes	− 0.23	0.28	0.65	0.419
Previous TIA/CVA	0.21	0.25	0.76	0.384
eGFR (mL/min/1.73m^2^)	0.00	0.01	0.07	0.789
Preoperative albumin (g/dL)	− 0.65	0.53	1.50	0.220
Number of RBC units transfused (u)	− 0.16	0.07	5.56	0.018
Intubation time (h)	− 0.05	0.02	4.64	0.031
Time < Hct^25^ (min)	− 0.01	0.00	5.88	0.015
Systolic blood pressure (mmHg)	0.01	0.01	1.22	0.269
Blood glucose (mg/dL)	− 0.01	0.01	1.73	0.189
Sodium (mEq/L)	− 0.00	0.05	0.00	0.991
Overall model fit		AIC = 244.1		0.003

Results of the multivariate ordered logistic regression are given as coefficient, standard error, and chi-square. As multivariate ordered logistic regression analyses for the ICDSC score, Model 1 incorporated nine significant variables to identify independent predictors of delirium, Model 2 incorporated time < Hct^25^ that was independent predictor in model 1 together with previously reported independent risk factors

## Discussion

In the present study, we investigated the risk factors for postoperative delirium in patients who underwent ODGP (maintaining DO_2_i > 300 mL/min/m^2^ through pump flow adjustments during CPB). Time < Hct^25^ was an independent predictor of the ICDSC score in model 1, and the number of RBC units transfused, intubation time, and time < Hct^25^ were independent predictors of the ICDSC score in model 2. Although patients with higher ICDSC scores had greater hemodilution during CPB, ODGP resulted in a higher PI, and DO_2_i was maintained above 300 mL/min/m^2^, with no significant difference between the three groups. These results suggest that the increase in pump flow due to ODGP could not compensate for the decreased Hct or prevent postoperative delirium. Further exploration is needed in larger populations to determine whether suppressing the decrease in Hct during CPB can prevent delirium following cardiac surgery.

Postoperative delirium is a common neurologic complication of cardiac surgery and is associated with short-term and long-term morbidity and mortality [2, 3]. While it is important to diagnose, treat, and manage delirium, it is also important to prevent its onset. In this study, the ICDSC score was used to divide the patients into three groups: non-delirium, subsyndromal delirium, and delirium. Subsyndromal delirium is an intermediate state between delirium and non-delirium, but the risk of developing delirium is high in this state, and the prognosis is also intermediate between delirium and non-delirium [18, 19]. The study reported that the ICU mortality rates were 2.4% in the non-delirium, 10.6% in the subsyndromal delirium, and 15.9% in the clinical delirium (*p* < 0.001, overall) [18]. Since the more delirium-related symptoms appear on the ICDSC, the higher the score; this may indicate not only the "presence" of delirium, but also the "severity" of delirium. Therefore, it is important to not only prevent the onset of delirium but also to suppress the symptoms of delirium.

Regarding preoperative risk factors, there was an association between BSA, diabetes, preoperative Hct, and Euro SCORE II and postoperative delirium, which is influenced by preoperative comorbidities. Low preoperative Hct, followed by low intraoperative Hct and number of RBC units transfused, were associated with postoperative delirium, suggesting the importance of perioperative hemodilution (Hct level) management. Hct during CPB tended to be lower in patients with higher ICDSC scores, and time < Hct^25^ was identified as an independent predictor of ICDSC score by two multivariate models. Perioperative low Hb or Hct and anemia have been identified as risk factors for delirium [20–22], but not in systematic reviews or meta-analyses [1, 23]. When evaluating hemodilution in cardiac surgery with CPB, it is generally necessary to analyze the lowest value during CPB or the difference between the preoperative value and the lowest value during CPB. The Hct or Hb level is dynamic, suggesting that analyzing data from a specific point in time cannot accurately evaluate hemodilution. Some studies [24, 25] have focused on the AUC to show the relationship between ScO_2_ and cognitive decline or delirium after cardiac surgery. The AUC of ScO_2_ accounts for both the depth and duration of oxygen debt below the threshold. Such results have presented both depth and duration of oxygen debt as the AUC rather than absolute values, which are more relevant in predicting organ ischemia. Therefore, the differences in the assessment of hemodilution during CPB (e.g., with or without AUC) might explain why variables related to hemodilution were identified or not identified as risk factors for delirium. We selected variables in the multivariate analysis based on time < Hct^25^ instead of AUC < Hct^25^ in this study because time < Hct^25^ was more significantly associated with delirium compared to nadir Hct, time < Hct^25^, and AUC < Hct^25^.

In the present study, there were no differences in the DO_2_ parameters between the three groups since ODGP maintained a DO_2_i > 300 mL/min/m^2^ through pump flow adjustments during CPB. Increased cumulative time and AUC below the DO_2_ thresholds have been noted to be independently associated with elevations in brain injury biomarker (ubiquitin C-terminal hydrolase L1) level [26]. Our preliminary results suggest the importance of optimizing DO_2_ management during CPB. A recent study reported that although DO_2_ parameters, such as AUC of DO_2_i, were clearly associated with delirium, DO_2_ could not be identified as an independent predictor of postoperative delirium [12]. However, unlike DO_2_ parameters, the evaluation of hemodilution was performed only at specific points in these studies. The lowest Hb on CPB just failed to reach significance but tended to be lower in patients with delirium (9.29 ± 1.27 vs. 8.89 ± 1.27; *p* = 0.053) [12]. It is likely that the hemodilution was not correctly assessed, and the results may have been different if a comprehensive method for assessing hemodilution, such as AUC, was used. A low Hct during CPB may reflect the severity of delirium, which cannot be modified by increasing the pump flow. Considering the two primary DO_2_ components, pump flow and Hb, as opposed to the increase in pump flow due to ODGP compensating for the decreased Hct and preventing postoperative AKI [13, 27], low Hct was an independent risk factor for delirium in this study. This indicates that the pump flow, Hb, or both should be adjusted to maintain DO_2_ during CPB depending on the target organs for oxygenation (e.g., renal oxygenation due to increased pump flow or cerebral oxygenation due to increased Hb, among others). The ability to prevent a low Hct during CPB might preclude the symptoms of delirium after cardiac surgery.

### Limitations

This study has some limitations. Because this relatively small study was conducted retrospectively, the measurement of variables may have been inconsistent and have selection and treatment bias, which may have influenced the analysis. Although evidence strongly suggests that previous TIA/CVA is an independent predictor of postoperative delirium in cardiac surgery, it was not identified as a risk factor in this study. The lack of consistency or reproducibility of risk factors indicates that the observed associations resulted from bias, confounding, or a statistical model. In this study, we emphasized the consideration of the two primary DO_2_ components, pump flow and Hct. Even though the sample size of the study is small, the low Hct during CPB still has an important predictive role in the development of postoperative delirium.

Other limitations of this study are the lack of known risk factors, such as reduced hearing, reduced visual acuity, comprehensive assessment of preoperative psycho-emotional status, cognitive status, and sleep deprivation. Moreover, the differences in ODGP, the assessment of hemodilution, or both may have been affected. Additionally, the small number of delirium occurrences limited our ability to develop comprehensive multivariable models. Thus, we considered the use of ICDSC to define postoperative delirium as appropriate; however, the ICDSC evaluation was performed within only the first 48 h postoperatively to minimize the influence of postoperative factors, which may have affected the present study.

### Conclusion

This study revealed that low Hct during CPB with ODGP, number of RBC units transfused, and intubation time were significantly associated with postoperative delirium following cardiac surgery. The efficacy of ODGP in increasing pump flow could not compensate for decreased Hct levels and prevent postoperative delirium. The ability of ODGP to increase Hct, which consequently prevents postoperative delirium, remains unproven. Further studies are needed to determine whether suppressing the decrease in Hct during CPB with ODGP can prevent delirium following cardiac surgery and to clarify the mechanism of delirium via low Hct following CPB.

## Data Availability

Data will not be shared because of institutional policy.

## Abbreviations

DO_2_: Oxygen delivery
CPB: Cardiopulmonary bypass
AKI: Acute kidney injury
ODGP: Oxygen delivery-guided perfusion
DO_2_i: Oxygen delivery index
eGFR: Estimated glomerular filtration rate
ICU: Intensive care unit
RBC: Red blood cell
MAP: Mean arterial pressure
TIA: Transient ischemic attack
CVA: Cerebral vascular accident
Hct: Hematocrit
AUC: Area under the curve
SaO_2_: Arterial oxygen saturation
PaO_2_: Arterial partial pressure of oxygen
BSA: Body surface area
PI: Perfusion index
Hb: Hemoglobin
ICDSC: Intensive Care Delirium Screening Checklist
ScO_2_: Cerebral oxygen saturation
SD: Standard deviation
ANOVA: Analysis of variance

## References

[1] Gosselt AN, Slooter AJ, Boere PR, Zaal IJ (2015). Risk factors for delirium after on-pump cardiac surgery: a systematic review. Crit Care.

[2] Mangusan RF, Hooper V, Denslow SA, Travis L (2015). Outcomes associated with postoperative delirium after cardiac surgery. Am J Crit Care.

[3] Martin BJ, Buth KJ, Arora RC, Baskett RJ (2012). Delirium: a cause for concern beyond the immediate postoperative period. Ann Thorac Surg.

[4] Saczynski JS, Marcantonio ER, Quach L, Fong TG, Gross A, Inouye SK, Jones RN (2012). Cognitive trajectories after postoperative delirium. N Engl J Med.

[5] Magruder JT, Dungan SP, Grimm JC, Harness HL, Wierschke C, Castillejo S (2015). Nadir oxygen delivery on bypass and hypotension increase acute kidney injury risk after cardiac operations. Ann Thorac Surg.

[6] Mukaida H, Matsushita S, Kuwaki K, Inotani T, Minami Y, Saigusa A (2019). Time-dose response of oxygen delivery during cardiopulmonary bypass predicts acute kidney injury. J Thorac Cardiovasc Surg.

[7] de Somer F, Mulholland JW, Bryan MR, Aloisio T, Van Nooten GJ, Ranucci M (2011). O_2_ delivery and CO_2_ production during cardiopulmonary bypass as determinants of acute kidney injury: time for a goal-directed perfusion management?. Crit Care.

[8] Magruder JT, Crawford TC, Harness HL, Grimm JC, Suarez-Pierre A, Wierschke C (2017). A pilot goal-directed perfusion initiative is associated with less acute kidney injury after cardiac surgery. J Thorac Cardiovasc Surg.

[9] Newland RF, Baker RA, Woodman RJ, Barnes MB, Willcox TW (2019). Australian and New Zealand collaborative perfusion registry. Predictive capacity of oxygen delivery during cardiopulmonary bypass on acute kidney injury. Ann Thorac Surg..

[10] Ranucci M, Johnson I, Willcox T, Baker RA, Boer C, Baumann A (2018). Goal-directed perfusion to reduce acute kidney injury: a randomized trial. J Thorac Cardiovasc Surg.

[11] Oshita T, Hiraoka A, Nakajima K, Muraki R, Arimichi M, Chikazawa G (2020). A better predictor of acute kidney injury after cardiac surgery: the largest area under the curve below the oxygen delivery threshold during cardiopulmonary bypass. J Am Heart Assoc.

[12] Leenders J, Overdevest E, van Straten B, Golab H (2018). The influence of oxygen delivery during cardiopulmonary bypass on the incidence of delirium in CABG patients; a retrospective study. Perfusion.

[13] Mukaida H, Matsushita S, Yamamoto T, Minami Y, Sato G, Asai T (2021). Oxygen delivery-guided perfusion for the prevention of acute kidney injury: A randomized controlled trial. J Thorac Cardiovasc Surg..

[14] Bergeron N, Dubois MJ, Dumont M, Dial S, Skrobik Y (2001). intensive care delirium screening checklist: evaluation of a new screening tool. Intensive Care Med.

[15] Hollinger A, Siegemund M, Goettel N, Steiner LA (2015). Postoperative delirium in cardiac surgery: an unavoidable menace?. J Cardiothorac Vasc Anesth.

[16] Ringaitienė D, Gineitytė D, Vicka V, Žvirblis T, Šipylaitė J, Irnius A (2015). Impact of malnutrition on postoperative delirium development after on pump coronary artery bypass grafting. J Cardiothorac Surg.

[17] Whitlock EL, Vannucci A, Avidan MS (2011). Postoperative delirium. Minerva Anestesiol.

[18] Ouimet S, Riker R, Bergeron N, Cossette M, Kavanagh B, Skrobik Y (2007). Subsyndromal delirium in the ICU: evidence for a disease spectrum. Intensive Care Med.

[19] Cole M, McCusker J, Dendukuri N, Han L (2003). The prognostic significance of subsyndromal delirium in elderly medical inpatients. J Am Geriatr Soc.

[20] Kazmierski J, Kowman M, Banach M, Fendler W, Okonski P, Banys A (2010). Incidence and predictors of delirium after cardiac surgery: results from The IPDACS Study. J Psychosom Res.

[21] Krzych LJ, Wybraniec MT, Krupka-Matuszczyk I, Skrzypek M, Bolkowska A, Wilczyński M (2013). Complex assessment of the incidence and risk factors of delirium in a large cohort of cardiac surgery patients: a single-center 6-year experience. Biomed Res Int.

[22] Tully PJ, Baker RA, Winefield HR, Turnbull DA (2010). Depression, anxiety disorders and type d personality as risk factors for delirium after cardiac surgery. Aust N Z J Psychiatry.

[23] Chen H, Mo L, Hu H, Ou Y, Luo J (2021). Risk factors of postoperative delirium after cardiac surgery: a meta-analysis. J Cardiothorac Surg.

[24] Slater JP, Guarino T, Stack J, Vinod K, Bustami RT, Brown JM (2009). Cerebral oxygen desaturation predicts cognitive decline and longer hospital stay after cardiac surgery. Ann Thorac Surg.

[25] Schoen J, Meyerrose J, Paarmann H, Heringlake M, Hueppe M, Berger KU (2011). Preoperative regional cerebral oxygen saturation is a predictor of postoperative delirium in on-pump cardiac surgery patients: a prospective observational trial. Crit Care.

[26] Magruder JT, Fraser CD, Grimm JC, Crawford TC, Beaty CA, Suarez-Pierre A (2018). Correlating oxygen delivery during cardiopulmonary bypass with the neurologic injury biomarker ubiquitin C-terminal hydrolase L1 (UCH-L1). J Cardiothorac Vasc Anesth.

[27] Magruder JT, Weiss SJ, DeAngelis KG, Haddle J, Desai ND, Szeto WY (2022). Correlating oxygen delivery on cardiopulmonary bypass with Society of Thoracic Surgeons outcomes following cardiac surgery. J Thorac Cardiovasc Surg..

